# Uridine affects amino acid metabolism in sow-piglets model and increases viability of pTr2 cells

**DOI:** 10.3389/fnut.2022.1018349

**Published:** 2022-10-20

**Authors:** Hong-ling Wang, Yilin Liu, Tiantian Zhou, Lumin Gao, Jianxi Li, Xin Wu, Yu-long Yin

**Affiliations:** ^1^Hunan Co-Innovation Center of Safety Animal Production, College of Animal Science and Technology, Hunan Agricultural University, Changsha, China; ^2^Henan Key Laboratory of Zhang Zhongjing Formulae and Herbs for Immunoregulation, Zhang Zhongjing College of Chinese Medicine, Nanyang Institute of Technology, Nanyang, China; ^3^CAS Key Laboratory of Agro-Ecological Processes in Subtropical Region, National Engineering Laboratory for Pollution Control and Waste Utilization in Livestock and Poultry Production, Hunan Provincial Engineering Research Center for Healthy Livestock and Poultry Production, Institute of Subtropical Agriculture, Chinese Academy of Sciences, Changsha, China; ^4^Jiangxi Agricultural Engineering College, Zhangshu, China

**Keywords:** uridine, reproductive performance, placental transport, amino acid, sows

## Abstract

**Background:**

As an important nucleoside precursor in salvage synthesis pathway of uridine monophosphate, uridine (UR) is the most abundant nucleotide in sow milk. This study aimed to investigate the effects of maternal UR supplementation during second trimester of gestation on reproductive performance and amino acid metabolism of Sows.

**Results:**

Results showed that compared to CON group, the average number of stillborn piglets per litter was significantly reduced (*P* < 0.05) with higher average piglet weight at birth in UR group (*P* = 0.083). Besides, dietary UR supplementation significantly increased TP in sow serum, BUN content in cord serum, and TP and ALB in newborn piglet serum (*P* < 0.05); but decreased AST level in sow serum and BUN level in piglet serum (*P* < 0.05). Importantly, free amino acids profile in sow serum newborn piglet serum and colostrum was changed by maternal UR supplementation during day 60 of pregnancy, as well as the expression of amino acids transporter (*P* < 0.05). In addition, from 100 to 2,000 μM UR can increased the viability of pTr2 cells. The UR exhibited higher distribution of G1/M phase of cell cycle at 400 μM compared with 0 μM, and reduced S-phases of cell cycle compared with 0 and 100μM (*P* < 0.05).

**Conclusion:**

Supplementation of uridine during day 60 of pregnancy can improve reproductive performance, regulate amino acid metabolism of sows and their offspring, and increase the viability of pTr2 cells.

## Introduction

The gastrointestinal tract and visceral organ and skeletal muscle protein synthesis of piglet are influenced by sow milk (1). Interestingly, in milk from various mammals, soluble nucleotides contribute to as much as 20% of its non-protein fraction (2). Exogenous nucleotides would increase nucleic acid and protein synthesis, promoting cell growth and proliferation under pathological conditions (3).

Attractively, the most abundant nucleotide is uridine monophosphate (UMP), which accounts for 86–98% of total 5′ nucleotides and has the highest content in sow’ colostrum (4). Therefore, UMP may be the most needful nucleotide not only for piglets but also for sows due to excessive secretion. UMP can be synthesized *in vivo*, but the synthesis of endogenous nucleotides from amino acids requires a great deal of energy (5). UR is an important nucleoside precursor in salvage synthesis pathway of UMP and most normal tissues rely on it for the recovery synthesis of pyrimidine nucleotides (6). Tissues could not synthesize uridine, therefore plasma uridine is used to maintain basic cellular functions in tissues (7). It was shown in our previous study that dietary UR bettered to improve the intestinal barrier and nucleotide transport in weaned piglets more than UMP (8).

A restricted feeding program is currently adopted by the swine industry to prevent excess weight gain during gestation, which can ease farrowing difficulties in gilts and sows, as well as appetite reduction of sows during lactation (9). However, sows need sufficient nutrients to support optimal fetal growth during gestation. Thus, accurate nutrition supply is very important for sows. In addition, the placenta regulates and supplies nutrient composition from mother to fetus and controls the source of hormonal signals that affect maternal and fetal metabolism (10). Consequently, the capacity of the placenta to transport nutrients is crucial to fetal growth. The specific transporter proteins in the plasma membrane of the syncytiotrophoblast are vital to the capacity of the placenta to transport nutrients (1). Our previous study showed that cytokine secretion and intestinal mucosal barrier function in suckling piglets could be regulated by maternal dietary supplementation with UR, leading to reduced incidence of diarrhea (11).

Therefore, the objectives of the present study were to explore the effects of maternal UR supplementation on reproductive performance of sows, serum biochemical parameters, serum amino acids profile of sow and piglet, placental amino acids transporter, and the growth of porcine trophectoderm cell.

## Materials and methods

### Ethics statement

The animal experiments were approved by the Institutional Animal Care and Use Committee of the Institute of Subtropical Agriculture, Chinese Academy of Sciences (2013020).

### Animals and experimental treatments

60 multiparous crossbred sows (Landrace × Yorkshire) were selected according to parity (parity 4–6), expected delivery and body shape, and divided into two groups which fed a basal diet (CON) or a basal diet with UR supplementation (120 g/t) from 60th day of pregnancy to delivery. All sows were fed twice daily at 06:30 and 15:00 h and had free access to water during the experiment period. Each sow was fed 2.5–3.0 kg/d from days 60 to 107 of pregnancy and approximately 1.8 kg/d on day 5 before delivery. Farrowing was not induced and was constantly supervised. Sows were identified by ear tags and the reproductive performance of sow was recorded at delivery. Newborn piglets were individually weighed, and IUGR piglet weigh approximately 618–869 g, defined as weighing approximately 65% of the birth weight of the largest littermate in each litter (12).

### Sample collection

After delivery, eight sows were randomly selected to collect blood sample in each group by the way of ear border vein in 5 ml vacuum tubes with one of them containing heparin lithium. The blood samples of eight neonatal piglets were collected (0800 h) before eating colostrum from jugular vein (13), and centrifuged for 15 min at 3,000 × g, 4^°^C, to obtain serum and plasma, and then stored at −20^°^C until analysis.

One allantochorion tissue sample (*n* = 8 sows/group) in the similar place of placenta (great vessels were avoided) of piglets with BW about 1.5 kg were obtained immediately after farrowing. Part of placenta was stored in liquid nitrogen for RT-qPCR analyses (14).

A total of 16 sows (Yorkshire × Landrace, *n* = 8 sows/group) were randomly selected to collect colostrum (30–40 mL/sow) from the 4–6th mammary glands of each sow within 12 h postpartum by massaging breasts (15). Colostrum was aliquoted and frozen at −70^°^C for 5 min, then stored at −20^°^C until it was assayed.

### Biochemical analyses

Automated Biochemistry Analyzer (Synchron CX Pro, Beckman Coulter, Fullerton, CA, USA) was used for analyze the concentrations of serum triglycerides (TG), total cholesterol (TC), high density lipoprotein (HDL), and low-density lipoprotein (LDL) according to the commercial kits and manufacturer’s instructions which was purchased from Beijing Chemlin Biotech Co., Ltd. (Beijing, China).

### Cell culture and treatment

The porcine trophectoderm cell line-2 (pTr2), which has been previously characterized and used for functional studies of porcine trophectoderm cell line, was originally isolated from the elongated porcine blastocysts, and collected on day 12 of pregnancy in this study (16). The cells (passages 30–35) were grown in 75 cm 2 flasks containing medium with 15 ml DMEM/F-12 with 5% FBS, 1% P/S, and 0.05% insulin, which was changed every 2 days. At confluence, the cells were collected using 0.125% trypsin solution (1.25 ml of 2.5% trypsin in 23.75 ml of 0.02% EDTA). After counting the number of cells, they were diluted to 4 × 104 cells/ml in DMEM/F-12 containing 5% FBS, 1% P/S, and 0.05% insulin.

### Cell counting kit-8

The pTr2 cells were subcultured in 96-well plates (30% confluence) in growth medium until the monolayer confluence reached 40% and then switched to custom medium free of serum and insulin. After starvation for 24 h, cells were added uridine (0, 50, 100, 200, 400, 600, 1,000, 1,500, and 2,000 μM) for 48 h to each well (*n* = 6 wells/treatment). Cells were treated with 10% CCK-8 (Dojindo; Kumamoto, Japan), diluted in normal culture medium at 37^°^C until visual color conversion occurred. OD (absorbance) values for each well were converted to relative cell number using standard curves.

### Cell cycle assay

The pTr2 cells were subcultured in 6-well plates (30% confluence) in medium until the monolayer confluence reached 40% and then switched to customized medium free of serum and insulin. After starvation for 24 h, cells were added uridine (0, 50, 100, 200, and 400 μM) for 48 h to each well (*n* = 3 wells/treatment). The cultured pTr2 cells were trypsinized and fixed in 75% cold alcohol overnight. The fixed cells were stained by propidium iodide (PI), and analyzed by flow cytometer (FACSCallbur, Becton-Dickinson). ModiFit software was used to analyze the proportion of cells in G0/G1, S, or G2/M phase.

### Ribonucleic acid extraction, reverse transcription and real-time PCR analysis

Total ribonucleic acid (RNA) was extracted from liver tissue using Trizol reagent (Beyotime Biotechnology, Shanghai, China). The total RNA was reverse transcribed into cDNA using reverse transcription kit from Takara Biomedical Technology, Japan. All primers were designed using Primer-BLAST on the National Center for Biotechnology Information (NCBI) website (Table 1). Using SYBR Green I Dye (Thermo fisher scientific, New York USA) according to the manufacturer’s instructions, Quantitative real-time RT-PCR (qRT-PCR) was performed using the lightcycler 480II real-time PCR system (Roche, Basel, Switzerland). The PCR cycling conditions were as follows: 95^°^C for 5 min, 98^°^C for 2min, followed by 40 cycles of 5 s at 98^°^C, 5 s at 60^°^C, 10 s at 95^°^C, and a final step of 1 s at 65^°^C. Gene expression analysis was performed by relative PCR amplification analysis (2–ΔΔCt).

**TABLE 1:** Primer used for real-time PCR.

Gene	Forward sequence (5′–3′)	Reverse sequence (5′–3′)
GLUT4	GGCCATCGTCATTGGCATTC	GTCAGGCGCTTCAGACTCTT
LAT1	TTTGTTATGCGGAACTGG	AAAGGTGATGGCAATGAC
LAT2	TCTCCATCCCACTGGTCACA	CGCTTCACATGGATCATGGC
SNAT1	AAGAACCTGGGCTATCTCGG	TGTTGCGTTAGGACTCGTTG
SNAT2	TACTTGGTTCTGCTGGTGTCC	GTTGTGGGCTGTGTAAAGGTG
ENT3	GGTTGGACTACGCCAGGTACT	GAGGGACTCGATGTTGGTGG
SANT4	GCTGTGGCAATCCTGTCACT	CCATCCAAATGCTTTTTCACCCA
VEGFA	GCCTTGCTGCTCTACCTCCA	TGGCGATGTTGAACTCCTCAGT
VEGFR2	GAGTGGCTCTGAGGAACGAG	ACACAACTCCATGCTGGTCA
EAAT1	GATGGGACCGCCCTCTAT	CGTGGCTGTGATGCTGATG
EAAT2	GGCTGCTGGACAGGATGA	TAAATGGACTGGGTCTTGGT
EAAT3	ATAGAAGTTGAAGACTGGGAAAT	GTGTTGCTGAACTGGAGGAG
PAT1	TGTGGACTTCTTCCTGATTGTC	CATTGTTGTGGCAGTTATTGGT
CAT-1	TGCCCATACTTCCCGTCC	GGTCCAGGTTACCGTCAG
PEPT1	CATCGCCATACCCTTCTG	TTCCCATCCATCGTGACATT
GAPDH	GTCTGGAGAAACCTGCCAAA	CCCTGTTGCTGTAGCCAAAT

### Statistical analysis

Data are expressed as the mean ± standard deviation of at least three independent experiments. The statistical significance between the two groups was assessed by the student’s *t*-test. In the cell experiment, Statistical significance was assessed by the one-way analysis of variance (ANOVA) followed by the Duncan test for multiple comparisons. Differences were considered statistically significant at *P* < 0.05. The IBM SPSS Statistics for Windows software, Version 22.0 (IBM Corp., Armonk, NY, USA), was used for statistical analyses.

## Results

### Effect of uridine on reproductive performance of sows

The reproduction performance of the sows was summarized in Table 2. The average number of born, live born, mummification, healthy, and IUGR piglets per litter was not affected by maternal UR supplement. Interestingly, the average number of stillborn piglets per litter was significantly reduced in UR group compared with CON group (*P* < 0.05). In addition, the average piglet weight at birth was higher in UR group than CON group (*P* = 0.083).

**TABLE 2:** Effect of UR on reproductive performance of sows.

Items	CON	UR	SEM	*P*-value
Total born piglets per litter, *n*	12.93	12.56	0.305	0.558
Live born piglets per litter, *n*	11.30	11.89	0.306	0.348
Stillborn piglets per litter, *n*	1.41^a^	0.50^b^	0.149	0.002
Mummification piglets per litter, *n*	0.26	0.22	0.101	0.860
Healthy piglets per litter, *n*	10.63	11.39	0.312	0.238
IUGR piglets per litter, *n*	0.78	0.56	0.152	0.480
Average weight of piglets at birth, kg	1.54	1.68	0.039	0.083

The a,b means columns with different letters are significantly different (*P* < 0.05), and columns with the same letters or no superscript are considered when *P* > 0.05. *n* = 8 replicates. The same below.

### Effects of uridine on serum biochemical parameters in sow serum, cord serum and piglet serum

The serum biochemical parameters in sow serum, cord serum and piglet serum were summarized in Table 3. Dietary UR supplementation significantly increased serum TP in sow serum (*P* < 0.05), but decreased AST level (*P* < 0.05). The BUN content in cord serum was significantly higher by maternal UR supplementation (*P* < 0.05). Meanwhile, the serum TP and ALB was significant increased, but the BUN level was decreased in piglet serum by UR administration during pregnancy (*P* < 0.05).

**TABLE 3:** Effect of UR on serum biochemical parameters in sow serum, cord serum and piglet serum.

Items	Sow serum				Cord serum				Piglet serum			
	CON group	UR group	SEM	*P*-value	CON group	UR group	SEM	*P*-value	CON group	UR group	SEM	*P*-value
TP (g/L)	71.91^b^	78.75^a^	1.70	0.039	22.65	23.86	1.63	0.725	24.00^b^	27.34^a^	0.87	0.050
ALB (g/L)	47.08	47.33	0.98	0.901	5.67	5.49	0.46	0.852	5.63^b^	6.87^a^	0.26	0.012
ALT (U/L)	34.67	35.4	1.58	0.824	6.51	6.18	0.65	0.807	15.51	18.67	1.24	0.216
AST (U/L)	45.89^a^	34.38^b^	2.81	0.036	23.14	23.57	2.17	0.926	47.29	45.38	2.63	0.731
ALP (U/L)	72.78	56.67	4.57	0.077	1826.63	1993.89	249.53	0.750	1794.38	1727.75	87.3	0.717
BUN mmol/L	4.29	3.81	0.24	0.335	5.79^b^	7.77^a^	0.39	0.007	4.24^a^	3.50^b^	0.16	0.018
GLU (mmol/L)	6.26	6.00	0.20	0.520	0.50	0.55	0.11	0.831	3.66	4.29	0.20	0.119

### Effects of uridine on free amino acids profile in sow and piglet serum and colostrum

Sows that were fed the UR-supplemented diet had lower serum contents of Ser and greater serum contents of Cys (*P* < 0.05, Table 4). Meanwhile, in piglets serum, the level of urea was significant decreased, but the Ile and Met level was increased by maternal UR administration during pregnancy (*P* < 0.05, Table 5). However, dietary UR supplementation significantly decreased Asp, Ser, Val, Leu, βAla, and His content in colostrum of sows (*P* < 0.05, Table 6).

**TABLE 4:** Effects of UR on free amino acids profile in sow serum.

Items (μg/ml)	CON	UR	*P*-value
Urea	4832.23 ± 239.87	4372.51 ± 438.48	0.375
Tau	242.87 ± 17.15	270.51 ± 27.99	0.427
Asp	34.68 ± 1.35	30.84 ± 3.79	0.364
Thr	296.56 ± 29.83	395.01 ± 46.45	0.094
Ser	210.06 ± 10.19^a^	180.21 ± 8.48^b^	0.042
Glu	603.63 ± 53.12	527.31 ± 47.78	0.301
Gly	999.69 ± 23.14	929.07 ± 78.74	0.411
Ala	1031.02 ± 60.45	957.57 ± 99	0.536
Val	416.13 ± 27.97	429.3 ± 19.63	0.705
Cys	51.04 ± 5.25^b^	98.94 ± 16.74^a^	0.022
Met	63.34 ± 4.56	73.52 ± 8.79	0.325
Ile	237.28 ± 16.55	227.51 ± 12.28	0.642
Leu	284.19 ± 23.78	276.9 ± 15.48	0.796
Tyr	357.08 ± 22.87	321.41 ± 19.63	0.254
Phe	263.07 ± 17.72	240.89 ± 10.57	0.298
Lys	373.84 ± 39.13	329.3 ± 36.21	0.416
His	178.28 ± 12.34	187.43 ± 12.63	0.611
Arg	411.95 ± 44.23	349.99 ± 27.55	0.267
Pro	570.23 ± 44.84	576.92 ± 66.45	0.935

**TABLE 5:** Effects of UR on free amino acids profile in piglet serum.

Items (μg/ml)	CON	UR	*P*-value
Urea	4907.43 ± 252.62^a^	3730.29 ± 163.77^b^	0.002
Tau	302.82 ± 51.67	268.42 ± 60.46	0.670
Asp	109.43 ± 15.37	107.52 ± 18.58	0.937
Thr	243.41 ± 32.66	262.34 ± 31.48	0.684
Ser	388.07 ± 30.53	385.57 ± 41.19	0.961
Glu	610.31 ± 108.5	797.73 ± 158.09	0.345
Gly	1223.72 ± 115.42	1005.21 ± 69.4	0.137
Ala	1313.72 ± 97.54	1225.06 ± 159.45	0.634
Cit	128.79 ± 10.23	110.75 ± 5.8	0.159
Val	443.74 ± 30.61	464.34 ± 41.25	0.694
Cys	112.19 ± 5.77	107.34 ± 10.15	0.684
Met	29.5 ± 2.79^b^	44.47 ± 6.4^a^	0.041
Ile	53 ± 4.22^b^	85.77 ± 12.87^a^	0.040
Leu	133.17 ± 8.49	148.38 ± 23.54	0.558
Tyr	263.86 ± 35.41	279.45 ± 33.93	0.756
Phe	154.03 ± 30.47	138.12 ± 14.75	0.658
Lys	630.95 ± 64.99	574.7 ± 92.69	0.627
His	107.43 ± 10.26	98.84 ± 10.7	0.571
Arg	106.31 ± 14.28	110.34 ± 14.68	0.847
Pro	344.97 ± 11.99	325.88 ± 33.58	0.601

**TABLE 6:** Effects of UR on free amino acids profile in colostrum.

Items (μg/ml)	CON	UR	*P*-value
Tau	3043.18 ± 507.8	2363.96 ± 166.43	0.155
Urea	3707.17 ± 250.42	3367.26 ± 397.54	0.482
Asp	12.76 ± 1.3	9.49 ± 0.81	0.051
Thr	28.71 ± 4.15	25.02 ± 2.94	0.479
Ser	24.86 ± 2.38 ^a^	17.71 ± 2.14 ^b^	0.040
Glu	84.7 ± 8.03	75.32 ± 6.93	0.389
Gly	12.15 ± 1.08	11.51 ± 1.85	0.769
Ala	30.03 ± 3.18	26.77 ± 2.28	0.418
Val	27.96 ± 4.59 ^a^	14.08 ± 1.94 ^b^	0.018
Cys	14.17 ± 2.79	10.28 ± 1.63	0.236
Ile	11 ± 2.56	7.2 ± 0.77	0.158
Leu	19.33 ± 2.82 ^a^	11.88 ± 1.82 ^b^	0.038
Tyr	18.21 ± 5.64	6.91 ± 0.97	0.137
Phe	18.28 ± 1.59	46.59 ± 19.94	0.194
βAla	80.85 ± 13.59^a^	40.02 ± 8.14^b^	0.025
Orn	4.85 ± 1.07	4.82 ± 0.76	0.985
Lys	21.85 ± 1.64	17.6 ± 2.45	0.182
His	9.01 ± 1.25^a^	4.85 ± 0.4	0.011
Arg	28.95 ± 2.7	26.17 ± 2.66	0.477
Pro	40.65 ± 5.01	37.91 ± 3.53	0.694

### Effects of uridine on amino acids transporter in the placenta of sows

**FIGURE 1:**
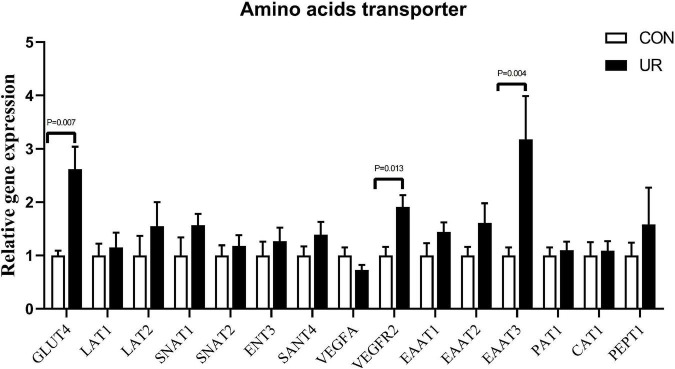
Effects of UR on amino acids transporter in the placenta of sows. The data was presented as mean ± SEM, significantly different was considered when *P* < 0.05, *n* = 6 replicates.

The expression of amino acids transporters in the placenta of sows was shown in Figure 1. Supplementing the maternal diet with additional UR significantly increased the mRNA expression of Glut4 and EAAT3 transporters in sow placentas (*P* < 0.05). In addition, a significant enhanced in VEGFR2 was also detected in sow placentas (*P* < 0.05).

### Effects of uridine on cell viability in the pTr2 cells

**FIGURE 2:**
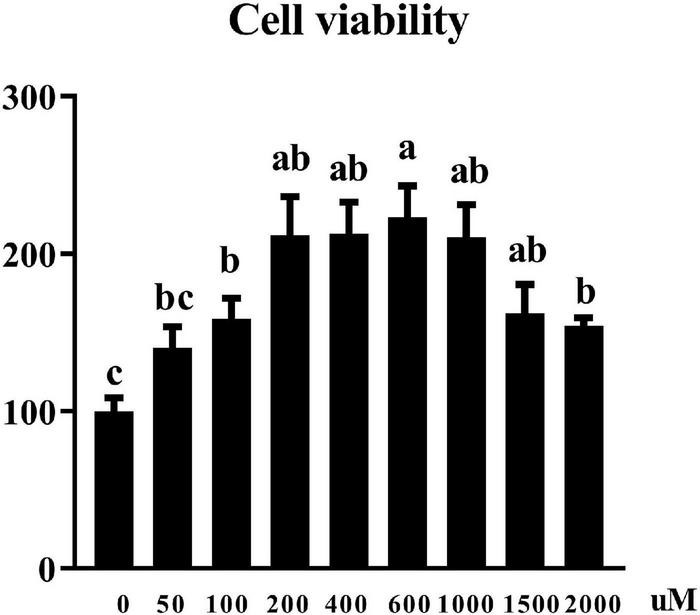
Effects of UR on cell viability in pTr2 cells. The a,b means columns with different letters are significantly different (*P* < 0.05), and columns with the same letters or no superscript are considered when *P* > 0.05. *n* = 6 replicates.

Cell viability was assessed by CCK8 assay. Figure 2 showed that UR at the concentration of 100–2,000 μM increased cell viability of pTr2 cells (*P* < 0.05). Importantly, cell viability is the highest at 200, 400, 600, and 1,000 μM of UR.

### Effects of uridine on cell cycle assay in the pTr2 cells

**FIGURE 3:**
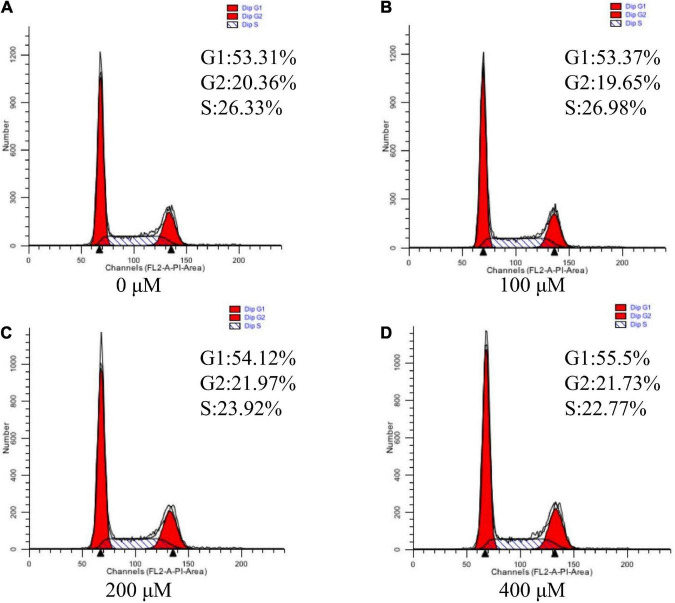
Effects of UR on cell cycle in pTr2 cells. Representative flow-cytometry diagrams of cells treated with 0 μM **(A)**, 100 μM **(B)**, 200 μM **(C)**, and 400 μM **(D)** UR for a 48-h 4-days period after starvation for 24 h.

Since cell cycle progression is also an important regulator of cell proliferation, we next explore whether UR regulated cell cycle (Figure 3). The results suggested that UR at 400 μM cells exhibited higher distribution of G1/M phase of cell cycle compared with 0 μM, and reduced S-phases of cell cycle compared with 0 and 100μM (*P* < 0.05, Table 7).

**TABLE 7:** Effects of UR on the distribution of cell cycle in pTr2 cells.

Items (%)	0 μM	100 μM	200 μM	400 μM	*P*-value
G1	53.8 ± 0.28^ab^	53.4 ± 0.26^b^	55.02 ± 1.05^ab^	55.72 ± 0.2^a^	0.066
G2	20.46 ± 0.36	20.92 ± 0.64	21.1 ± 0.62	21.91 ± 0.42	0.326
S	25.75 ± 0.57^a^	25.67 ± 0.71^a^	23.89 ± 1.39^ab^	22.37 ± 0.61^b^	0.077

The a,b means columns with different letters are significantly different (*P* < 0.05), and columns with the same letters or no superscript are considered when *P* > 0.05. *n* = 3 replicates.

## Discussion

Maternal nutrient supply may act as an inducer of fetal development and can permanently affects anatomy, physiology, and metabolism of fetus (17). Meanwhile, dietary nucleotides are non-protein nitrogenous compounds that modulate protein synthesis and is important for growth, repair, and differentiation of body tissue, including liver and small intestine (18). Dietary nucleotides supplement increased ADFI of weaned pigs and ADG, ADFI, and G: F of nursery pigs (19, 20). Consistently, our previous studies also showed UR supplement promoted nucleotide transport in weaned piglets and improved the growth performance in early weaned piglets (8, 21). Attractively, IUGR pig had improved nutrients utilization, intestinal function and immunity as a result of dietary nucleotides supplementation (22). Pregnancy is a critical period for fetal growth and development, however, there no research about dietary nucleotides supplement in sows that restricted feeding during gestation and excessive secreted milk during gestation. The results from the present study showed that UR supplement in sow diet during pregnant period decreased the number of stillborn piglets per litter and enhanced piglet weight at birth by 9.09%.

Maximizing placental protein synthesis and placental growth (including vascular growth) is related to maternal amino acid metabolism (23). Nucleotides modulate protein synthesis in the liver, and improve nutrients utilization (18, 22). TP indicates the body’s nutritional status, protein absorption, and metabolism, while BUN indicates protein degradation (24). In the present study, the results showed that UR supplementation during the gestation period increased serum TP content of both sows and piglets and deceased serum BUN content, which may suggest UR improved the protein metabolism. Similarly, nucleotide-supplemented augmented the process of protein synthesis in liver injury rats (3). Piglets receiving diet with nucleotides additives experienced an accelerated carbon turnover during the post-weaning period (25). In addition, one-carbon metabolism is an essential material for the synthesis of purines, dTMP, and methionine (26). Addition of yeast RNA contributed to significantly reversing the effects of folate/methyl deficiency (27). In present study, UR supplement significantly affected the profile of serine and cysteine in sow serum, as well as the profile of methionine in piglet serum. It demonstrated that UR can improve methionine by affecting one-carbon metabolism both in sows and piglets mediated by placenta transmission. Moreover, nucleotides can alleviate the decrease of leucine concentration in the liver caused by alcoholic liver injury (28). However, our results showed no effect on the leucine content which may due to the different animal model. Moreover, maternal amino acid metabolism plays an important role in maximizing placental protein synthesis and placental growth (including vascular growth) (23). The insulin independent 1 (GLUT1) and insulin-dependent 4 (GLUT4) glucose transporters could regulate the entry of glucose into the adipocyte for *de novo* fatty acid and triglyceride synthesis, vital for the lipogenesis (29). It indicates the changes of glucose metabolism in sow’s placenta. In this experiment, EATT3 gene expression also changed, which is the excitatory amino acid transporters family, but the content of glutamate in sow and piglet serum and colostrum did not change. It may due to the glutamate transporter EAAT3 regulates extracellular glutamate levels, but its contribution to glutamate uptake is much lower than that of the astrocytic transporters EAAT1 and EAAT2 (30).

The size, morphology, and nutrient transfer capacity of placenta plays a direct decision role to the prenatal growth trajectory of the fetus, which further influence the birth weight (31), because of placenta acting as the key organ for the exchange of oxygen, nutrients, and wastes between the maternal-fetal circulations (32). Vasculogenesis is *de novo* formation of blood vessels from mesoderm precursor cells, and angiogenesis is creation of new vessels from a preexisting blood supply, which is very important for the maternal-fetal exchange (31). In the Yorkshire pig, fetal growth from mid to late gestation is associated with an increase in area of endometrial attachment (placental exchange area) (10). In present study, uridine can increase the relative mRNA expression of VEGFR gene in placenta, so as to increase the birth weight of piglets. In pigs, the elongation of placenta is the main factor determining the size of placenta. Around the 13th day of pregnancy, the pig toe nail began to attach to the uterine epithelium, more stable adhesion was carried out on the 15th–16th day, and formal implantation was carried out on the 18th–30th day (33). The blastocyst is composed of two distinct cell layers, with the trophectoderm accounting for two thirds of the cells (16, 34). The *in vitro* experiments also showed that uridine at an appropriate concentration can improve cell viability and cell cycle of pTr2 cells, which suggested the vital role of uridine in pTr2 cell proliferation.

## Conclusion

The supplement of uridine at day 60 of pregnancy reduced the average number of stillborn piglets per litter with higher average piglet weight at birth, and UR may affect the growth of placental tissue by increasing the viability of pTr2 cells, and the amino acid metabolism both of sows and piglets.

## Data availability statement

The datasets used during the study are available in the figshare repository, accession numbers doi: 10.6084/m9.figshare.21324357; doi: 10.6084/m9.figshare.21324366; and doi: 10.6084/m9.figshare.21324384.

## Ethics statement

This animal study was reviewed and approved by the Institutional Animal Care and Use Committee of the Institute of Subtropical Agriculture, Chinese Academy of Sciences (2013020).

## Author contributions

XW and Y-LY: conceptualization. YL: data curation and writing—original draft. H-LW, LG, and TZ: formal analysis. H-LW and YL: project administration. JL: resources. H-LW, LG, TZ, and XW: writing—review and editing. All authors contributed to the article and approved the submitted version.
